# A new 3D full-body scanner analyzing the sagittal and coronal balance of the adult spine: a preliminary prospective observational study

**DOI:** 10.1007/s00701-024-06411-5

**Published:** 2025-01-24

**Authors:** Tae Hoon Kang, Seokin Jang, Inwook Seo, Minseok Choi, Yongsoo Park, Yohan Lee, Jae Hyup Lee, Minjoon Cho

**Affiliations:** 1 Department of Orthopaedic Surgery, Seoul National University College of Medicine, SMG-SNU Boramae Medical Center, 20 Boramae-Ro 5-Gil, Dongjak-Gu, Seoul, Republic of Korea; 2 Medi Help Line Co, Ltd, 131, Toegye-ro, Jung-gu, Seoul, South Korea

**Keywords:** Sagittal and coronal balance of the adult spine, 3D full body scanner, Anthropometry, Adult spine deformity, Compensatory mechanism of positive sagittal balance, Dynamic spine balance

## Abstract

**Background:**

The degenerative spondylosis can cause the difficulty in maintaining sagittal and coronal alignment of spine, and X-ray parameters are the gold standard to analyze the malalignment. This study aimed to develop a new 3D full body scanner to analyze the spinal balance and compare it to X-ray parameters.

**Methods:**

Ninety-seven adult participants who suffer degenerative spondylosis underwent 3D full body scanning, whole spine X-rays, clinical questionnaires and body composition analyses. The 5 inflection points (ear, shoulder, hip, knee, ankle) of the 3D scanner in the sagittal plane were automatically labeled by an AI algorithm. Three concepts are created including “the angle between two points with respect to the plumb line”, “the horizontal distances between two points in the sagittal plane” and “the angle between three points”. For the coronal plane, the shoulder gradient was analyzed. X-ray parameters of cervical, thoracolumbar and whole spine sagittal balance and coronal balances were compared. The body composition data and clinical questionnaire scores were compared to x-ray and 3D scanner parameters.

**Results:**

The correlation coefficient (C.C.) of dAB_hor (horizontal distance between ear and shoulder in the sagittal plane) and C2-C7 SVA was 0.478 (*p*-value < 0.001). The C.C. of aAC_sag (sagittal angle of ear-hip from the plumb line) and ODHA was 0.336 (*p* < 0.001). About coronal balance, the C.C. of shoulder gradient and clavicle angle from x-ray was 0.373 (*p* < 0.001). The C.C.s were merely affected by body composition data. But in multiple regression analysis, BMI affected 3D scanner data. Clinical symptoms showed correlations with aBCD(shoulder-hip-knee) and aCDE(hip-knee-ankle angle), which may reflect a compensatory pelvic retroversion and knee flexion for positive sagittal imbalance.

**Conclusions:**

This new 3D scanner has some strengths like radiation-free methods, correlation with x-ray parameters and clinical symptoms, independence to body composition data, and possibility of analyzing dynamic spine balance.

## Introduction

As humans stand against gravity, the spines should keep the balance in the sagittal and coronal plane, because the mechanical balance fulfills the minimal energy consumption, which is called the concept of “Within a cone of equilibrium” [15]. However, the degenerative spondylosis, the degenerative change of adult spines, causes a malalignment and finally the enlarged energy consumption leads clinical symptoms like pains or discomfort of daily livings [12, 25]. The adult spine deformity (ASD), the malalignment of degenerative spondylosis is becoming increasingly common with the aging population [6].

Therefore, numerous studies presented the method of analyzing the balance of the spine. Until now, X-ray parameters in the sagittal and coronal planes are the gold standard [6, 29]. For examples, there are various parameters including global sagittal parameters like sagittal vertical axis (SVA) and spinopelvic parameters such as pelvic incidence (PI). However, conventional X-ray images have some limitations like radiation hazards and two-dimensional images. To overcome those issues, new devices like the EOS system (EOS® imaging, Paris, France) have been developed, which use ultra-low radiation doses to capture planar X-ray images and reconstruct 3D images [7, 17]. However, due to the EOS system’s high setup and maintenance costs, it may not be suitable for small clinics or non-medical institutions [7].

On the other hand, with the advancement of optical technology and the reduction in the cost of imaging equipment, 3D scanning devices have been developed in many parts [16]. The 3D body scanning devices have been developed in medical instruments because they could escape the radiation hazard and some studies tried to figure out the discrepancy of conventional anthropometrics and 3D scanners [34]. About analyzing the structure and balance of the spine, many studies have focused on adolescent idiopathic scoliosis (AIS) affecting children and adolescents [11, 13, 14, 33]. Because, AIS patients are young aged who are vulnerable to radiation exposure and also serial x-ray images are required during growing up [21]. So many studies tried to build a 3D body scanner and compare it with conventional x-rays to replace the need of radiation.

Meanwhile, to diagnose and follow up the malalignment of the degenerative spine, x-ray images are also needed. And multiple exposures to radiation cause a hazard even for adults and there were many trials to reduce the radiation exposure [10, 23]. Likewise, there were some studies of 3D scanners analyzing adult spines [28]. However, ASD itself is very hard to be diagnosed by 3D body scanners. The elder persons tend to have diverse body contours and numerous body composition status like obesity or sarcopenia. Previous studies showed the body composition data could affect the discrepancy between 3D scanner and X-ray measurements [11, 28]. And the ASD is also associated with chronic back pain and discomfort in daily life more than AIS, which results in the importance of correlation between clinical symptoms and ASD parameters [4, 25]. Therefore, prior 3D full-body scanner methods for AIS have limitations in understanding ASD [33].

We were interested in developing a new 3D full body scanner to analyze the balance of adult spines. After getting 3D full body scanner data, we need a new system to interpret them to sagittal and coronal balance data. After that, we planned to assess the efficacy by comparing with X-ray parameters. And considering the characteristics of degenerative spondylosis, we planned to figure out the effects of body composition data to 3D scanner data and the correlation of 3D data with clinical symptoms. Our first hypothesis is that our new 3D full body scanner and data processing method could fulfill the analysis of the adult spine alignment. And the second one is that 3D full body scanner data are independent from the body composition and has some correlations with clinical symptoms.

## Methods

### Device information

The 3D full-body scanner was MediAvatar from Medi Help Line, a corporation of Republic of Korea (ROK). This device comprised a multi-connected (3) RealSense D415 Depth Camera Module of Intel. This device has a certificate of patent in the ROK (10–2570452) and is qualified as a human anthropometric device by the government of ROK.

### Study design, materials, inclusion and exclusion criteria

This study design is a prospective observational study. As it is a preliminary study to analyze various status of adult spine balance, we planned to enroll patients who have clinical symptoms.

The first inclusion criterion is that the participant would be an adult (older than 20 years old). And the second one is that the participant came to our clinics for backaches at first time. Third one was that the doctors diagnosed the clinical symptoms were came from the lumbar spondylosis by x-ray images. There were some exclusion criteria. First, we excluded the patients with more than Koval grade 4 and limitation of self-standing [24]. Because they have difficulty standing alone and have a high risk of falling during the 3D full-body scanning. And also, we excluded the pregnant women and patients with difficulties in understanding and answering questionnaires.

From October 2022 to August 2023, more than 1000 patients came to our clinics. There were 181 participants who agreed and underwent the 3D body scanning. But only 96 patients fulfilled the clinical questionnaires, whole spine x-rays and body composition studies without loss. The final number of participants was 96.

### Demographic factors, x-ray parameters, clinical questionnaire, and body composition analysis

We checked the demographic factors, including gender, age, height, and weight.

About x-ray, both anteroposterior (AP) and lateral views of the whole spine were gained as a routine way in our hospital. Participants stood alone with their hands on their shoulders to show vertebral bodies well in the lateral X-ray view. We chose C2-7 sagittal vertical axis (SVA) and T1 slope as cervical sagittal parameters, SVA as thoracolumbar sagittal parameters, and Odontoid Hip Axis angle (ODHA) as global sagittal parameter from cervical to hip joints**.** We also checked the lumbopelvic parameters, including the pelvic incidence (PI), pelvic tilt (PT), sacral slope (SS), lumbar lordosis (LL), and PI-LL mismatch. In AP view, we checked clavicle angle and T1 tilt angle.^11,13–15^

Clinical questionnaires included the visual analog scale (VAS, 0–10) score of low back pain and the modified Oswestry Disability Index (mODI, 0–45) about the activity interference. Because mODI was related to compensatory failure to the spinal balance due to lumbar spondylosis and ASD [35].

We checked body composition data such as body mass index (BMI), percentage body fat (PBF), and skeletal muscle index (SMI) using a body composition analyzer. (ACCUNIQ BC380, SELVAS, South Korea) (Table 1).

**Table 1:** Demographic and X-ray measurement

Demographic factors	Mean ± standard deviation / *N* (%)	Radiologic data	Mean ± standard deviation / *N* (%)
Age	65.3 ± 9.5	*Cervical sagittal parameter*	
Sex, Male: Female	38 (39.2): 59 (60.8)	C2-7 SVA (mm)	19.2 ± 11.1
Height (cm)	162.4 ± 7.1	T1 slope (°)	24.7 ± 8.7
Weight (Kg)	65.3 ± 10.0	*Thoracolumbar sagittal parameter*	
*Body composition data*		SVA (mm)	21.5 ± 27.4
BMI(Kg/m^2^)	24.8 ± 3.3	*Global sagittal parameter*	
BMI group, < 25: ≥ 25	57 (59.4): 34 (35.4)	ODHA (°)	−1.4 ± 2.7
PBF(%)	30.2 ± 8.0	*Lumbopelvic parameter*	
SMI(%)	6.9 ± 0.9	Pelvic incidence (°)	48.7 ± 8.7
		Sacral slope (°)	31.0 ± 7.1
		Pelvic tilt (°)	17.7 ± 7.8
		Lumbar lordosis (°)	42.0 ± 11.9
		PI-LL mismatch (°)	2.9 ± 6.6
		*Coronal parameter*	
		Clavicle angle (°)	−0.04 ± 2.20
		T1 coronal tilt (°)	0.62 ± 4.31

*BMI *Body mass index, *PBF* Percentage body fat, *SMI* Skeletal muscle index, *SVA* Sagittal vertical axis, *ODHA *Odontoid hip axis angle, *PI-LL* mismatch Pelvic incidence – Lumbar lordosis mismatch

### 3D scanner measurement

Participants were given well-fitting clothing, and privacy screens were used. We instructed them to stand in a natural position while holding handles. The height of the handles is adjustable, and it was impossible to lean their upper body on the handles. The 3D scanning device utilizes infrared rays to image the body surface and make 3D rendering images. The time of one rotating scan is 45 s and the time of data processing is 3 min. To minimize the possibility of errors in data collection and processing, we conducted rotating scans at least 2 times for each participant. And also, the medical doctor and engineer of the scanner checked the data simultaneously and figured out if an error happened. If there was error data, a new scanning was done. To ensure the accuracy of the results in repeated measurements, we calculated the ICC (intraclass correlation coefficient) which was 0.93. Therefore, we used the mean values of more than two scans.

The software automatically recognizes the 3D images and coordinates by measuring the inflection points using AI algorithm analysis (3D Human model Feature Point Recognition algorithm, the project “Size Korea”, ROK). This AI algorithm is a revised version of the human body landmark labeling AI algorithm of the government project of ROK based on ten thousand human body scan data. (ISO 18825–2, KS A ISO 7250–1:2016) In the sagittal plane analysis, points A(the center of ear), B(the lateral side of shoulder), C(the greater trochanter of femur), D(the lateral side of the knee), and E(the lateral malleolus of ankle) were recognized automatically and labeled. The expert double checked whether the error data occurred or not.

**Fig. 1:**
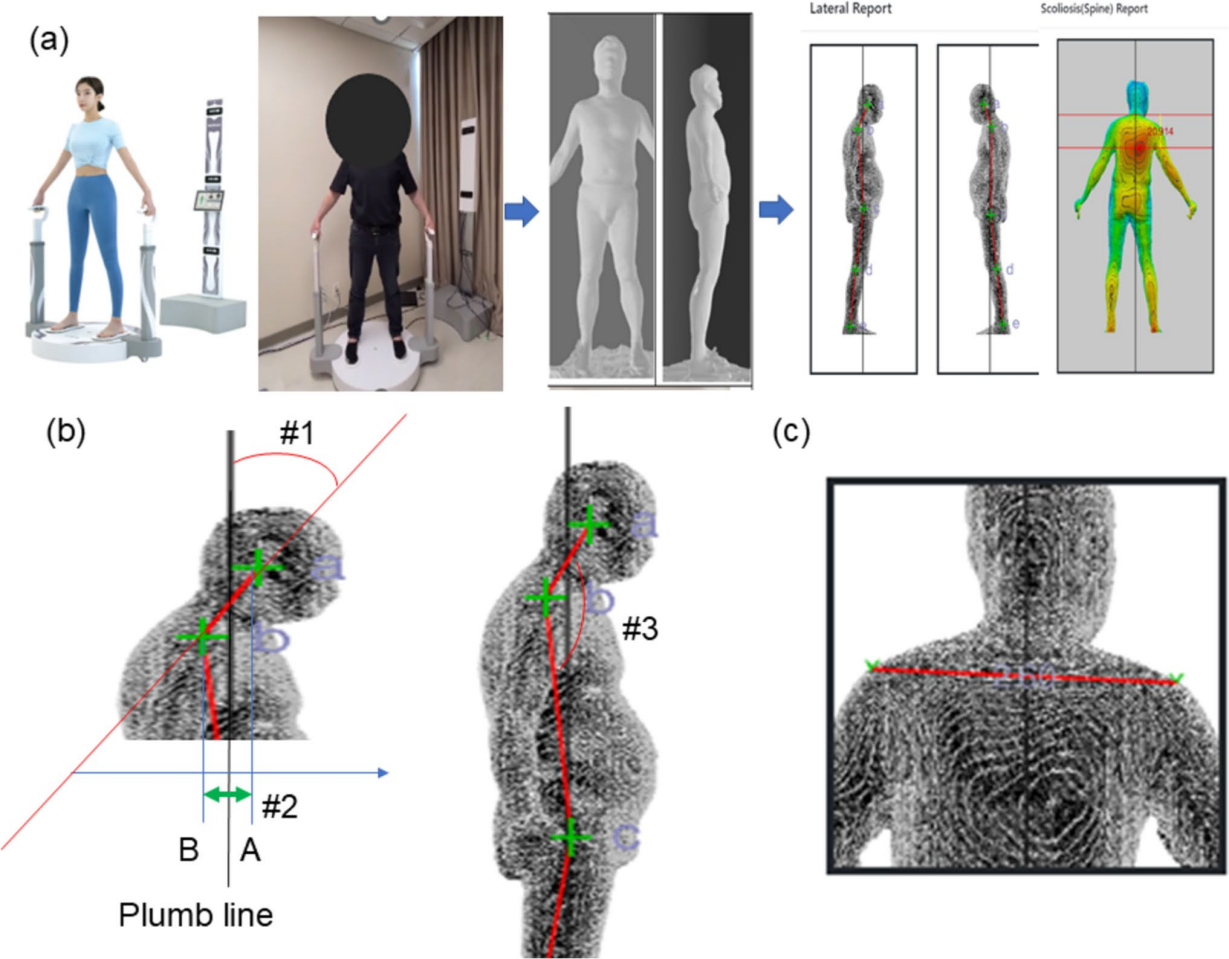
** a** Full sequence of 3D scanner measurement, 3D surfacing technique, and automated labeling of inflection points. **b** Three concepts to measure sagittal balance. #1. The angle of two points with respect to the plumb line ex) aAB_sag / #2. The horizontal distance between two points ex) dAB_hor / #3. The angle of three points ex) aABC **c** Shoulder gradient to measure coronal balance

To alter the 3D scanner data to sagittal balance information, we created three concepts to analyze the correlation between the points. The first one is the angle of two points with respect to the plumb line (ex. aAB_sag). The second one is the horizontal distance between two points in the sagittal plane (ex. dAB_hor). The third one is the angle between three points (ex. aABC). Inevitably, the data were formed in two different planes (the left side and right side). We used the mean value of each parameter from the different planes. In the coronal plane analysis, shoulder gradients were recognized automatically and labeled. Additionally, we obtained the waist circumference, hip circumference, and waist-hip ratio from 3D scanning data (Table 2) (Fig. 1).

**Table 2:** 3 D full-body scanner data

	Mean ± standard deviation
Sagittal	
aAB_sag(°)	13.4 ± 9.9
aBC_sag(°)	−2.5 ± 4.4
aAC_sag(°)	1.3 ± 4.4
dAB_hor(mm)	37.4 ± 25.7
dBC_hor(mm)	−22.8 ± 36.7
dAC_hor(mm)	14.6 ± 46.7
aABC(related to neck flexion) (°)	168.5 ± 12.9
aBCD(related to hip flexion) (°)	187.1 ± 6.7
aCDE(related to knee flexion) (°)	185.5 ± 4.9
Coronal	
Shoulder gradient (Lt. +)	2.1 ± 2.2
Body circumference	
Waist circumference(cm)	98.76 ± 17.32
Hip circumference(cm)	101.93 ± 15.61
Waist/hip ratio	0.97 ± 0.15

*aAB_sag* Angle of AB with respect to plumb line, kyphosis + , *dAB_hor *Horizontal distance between A and B, kyphosis + , *aABC* Angle of ABC

### Data collection and statistical analysis

Two orthopedic doctors (K.T.H. and C.M.S) measured parameters in whole spine x-ray images. The X-ray images were viewed on a PACS viewer (PACS M6, INFINITT Healthcare, South Korea). The ICC between two observers was 0.96, and we used the mean values. Correlation analyses were performed (Continuous variables as Pearson correlation coefficient; Ordinal variables as Spearman’s correlation coefficient). To evaluate the effect of body composition between 3D scanner and x-ray, partial correlation coefficients adjusting body composition factors were analyzed. Simple and multiple linear regression analyses were performed to assess the linear relationship of 3D scanner data with other factors. All data were analyzed using Rex pro 3.6.0 (Rexsoft, South Korea).

## Results

### Correlation coefficient for the cervical sagittal balance (Fig. 2)

**Fig. 2:**
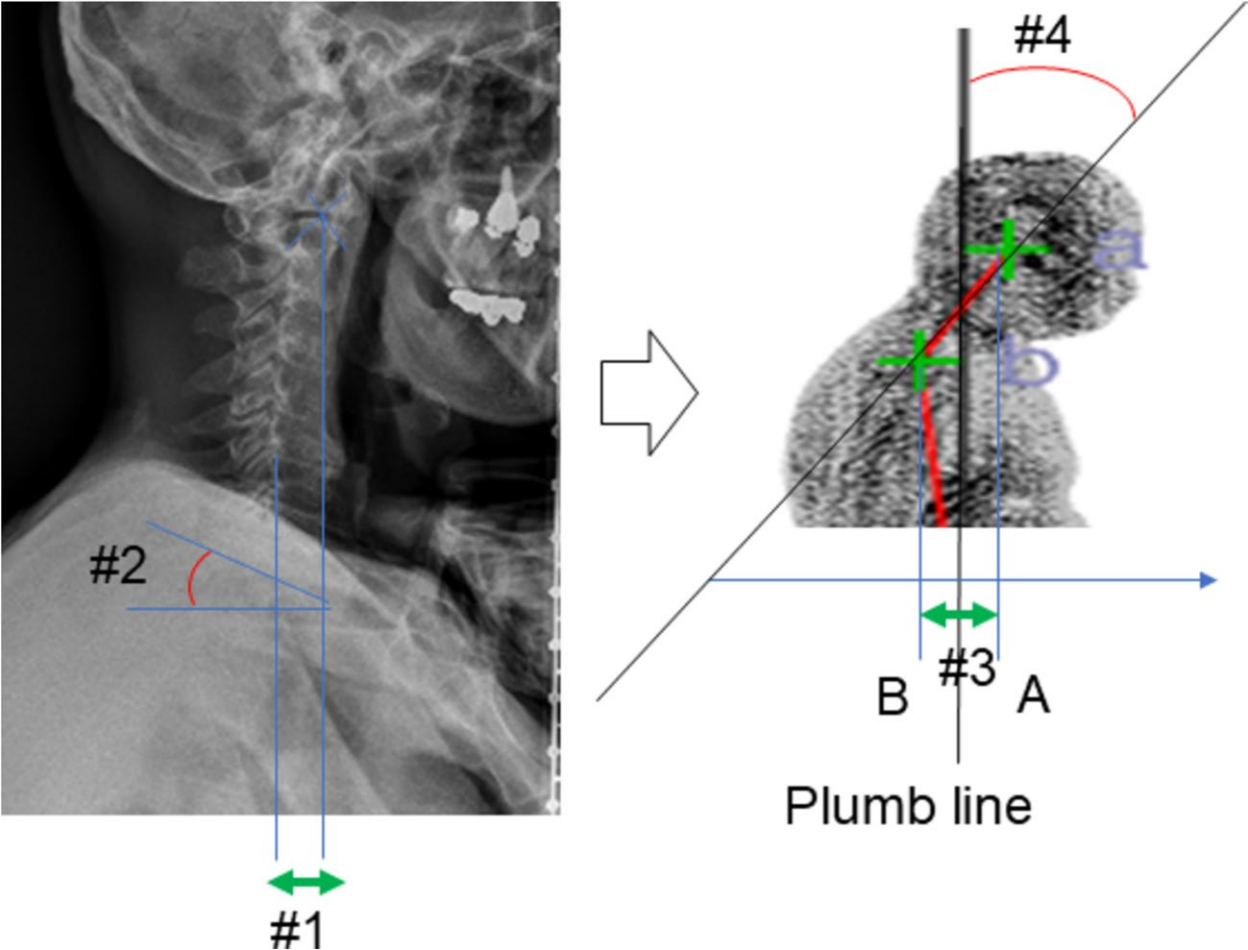
#1. C2-7SVA / #2. T1 slope on x-ray / #3. dAB_hor / #4. aAB_sag on the 3D scanner

The aAB_sag showed a correlation with cervical kyphosis parameters (C2-7 SVA, T1 slope). The correlation coefficient (C.C.) and *p*-value were 0.496 and < 0.001 for the C2-7 SVA, and 0.281 and 0.005 for the T1 slope. Similarly, the dAB_hor showed a correlation with them. The C.C. and *p*-value were 0.478 and < 0.001 for the C2-7 SVA, and 0.276 and 0.006 for the T1 slope.

### Correlation coefficient for the thoracolumbar sagittal balance (Fig. 3)

**Fig. 3:**
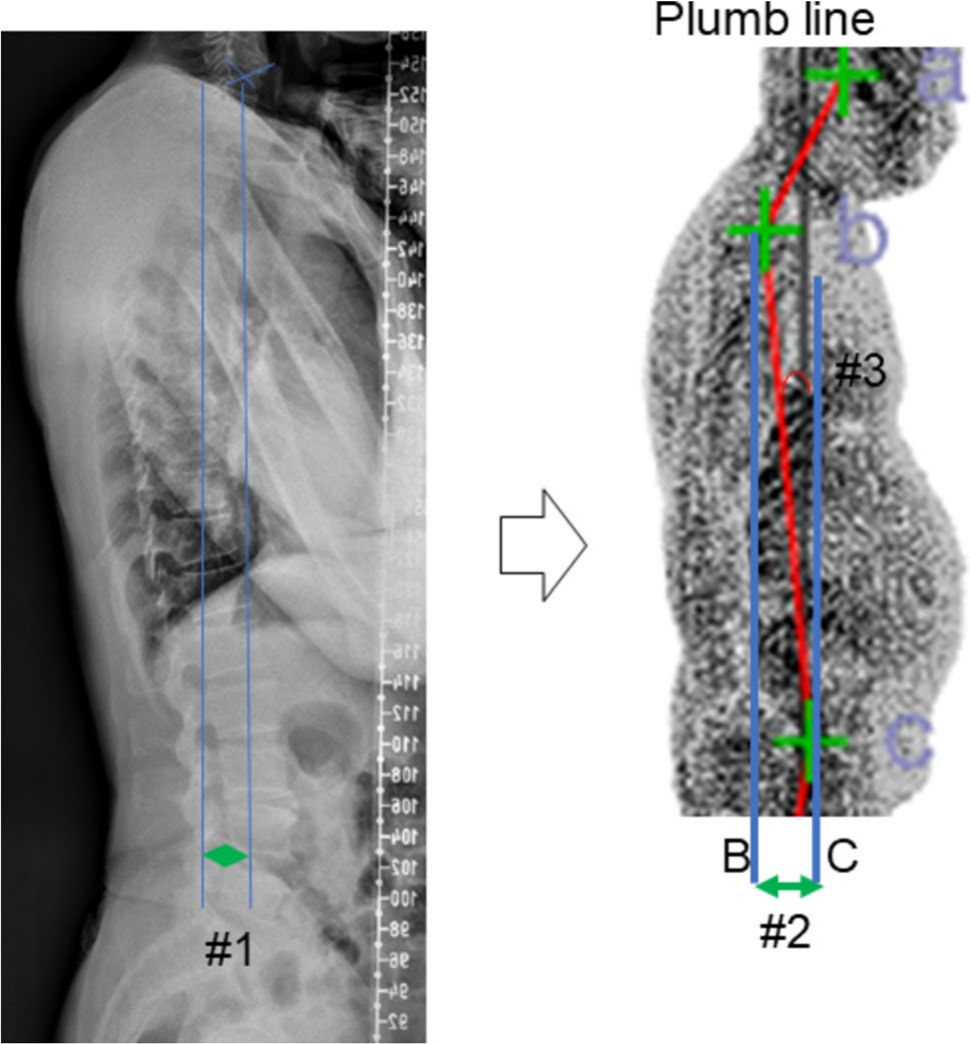
#1. SVA on x-ray / #2. dBC_hor / #3. aBC_sag on the 3D scanner

The aBC_sag was correlated with SVA, with C.C. and *p*-value of 0.222 and 0.029. Also, the dBC_hor was related to SVA, and the C.C. and *p*-value were 0.223 and 0.028.

### Correlation coefficient for the global sagittal balance (Fig. 4)

**Fig. 4:**
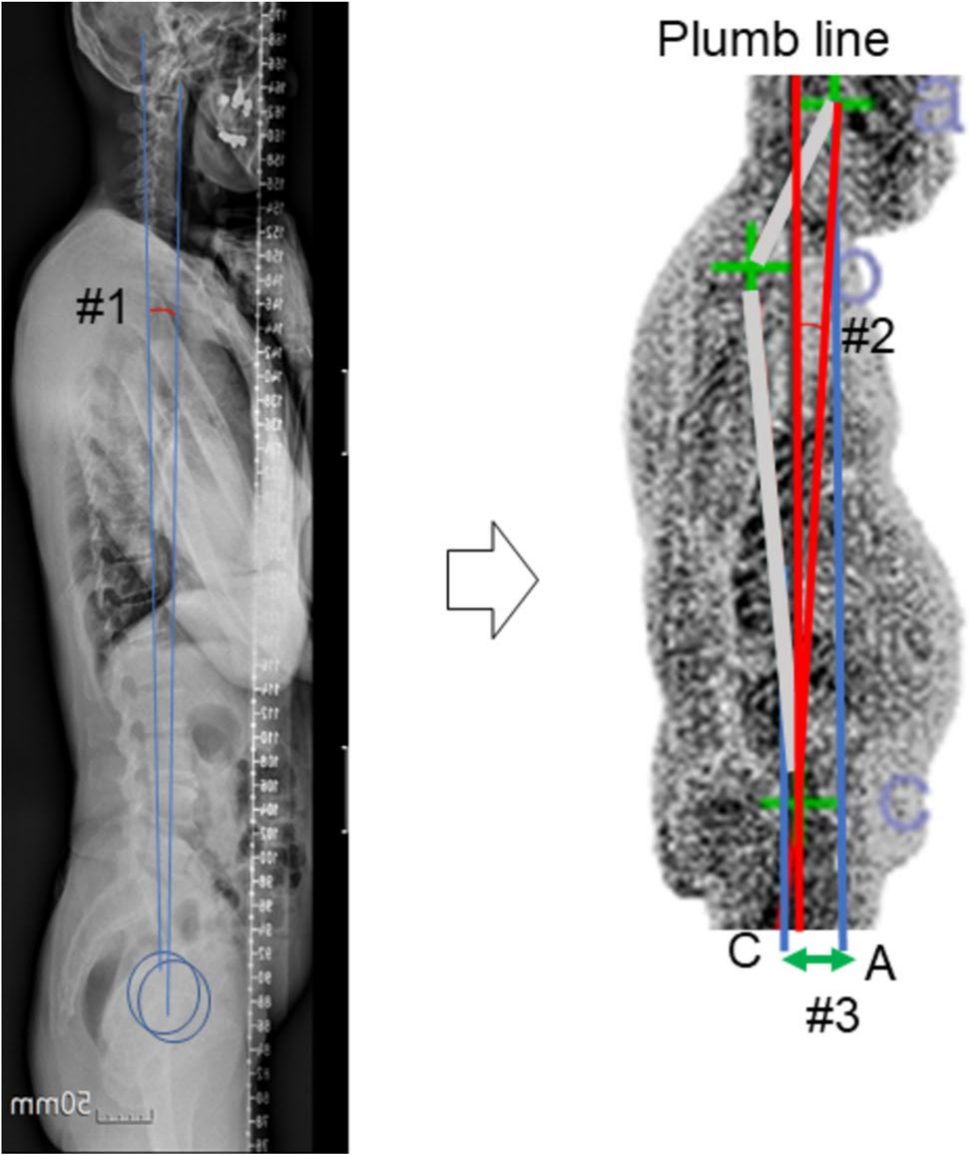
#1. ODHA on x-ray / #2. aAC_sag / #3. dAC_hor on the 3D scanner

The aAC_sag showed a correlation with ODHA. The C.C. and *p*-value were 0.336 and < 0.001. Likewise, the dAC_hor was related to ODHA, and the C.C. and *p*-value were 0.335 and < 0.001.

### Correlation coefficient for the coronal balance (Fig. 5)

**Fig. 5:**
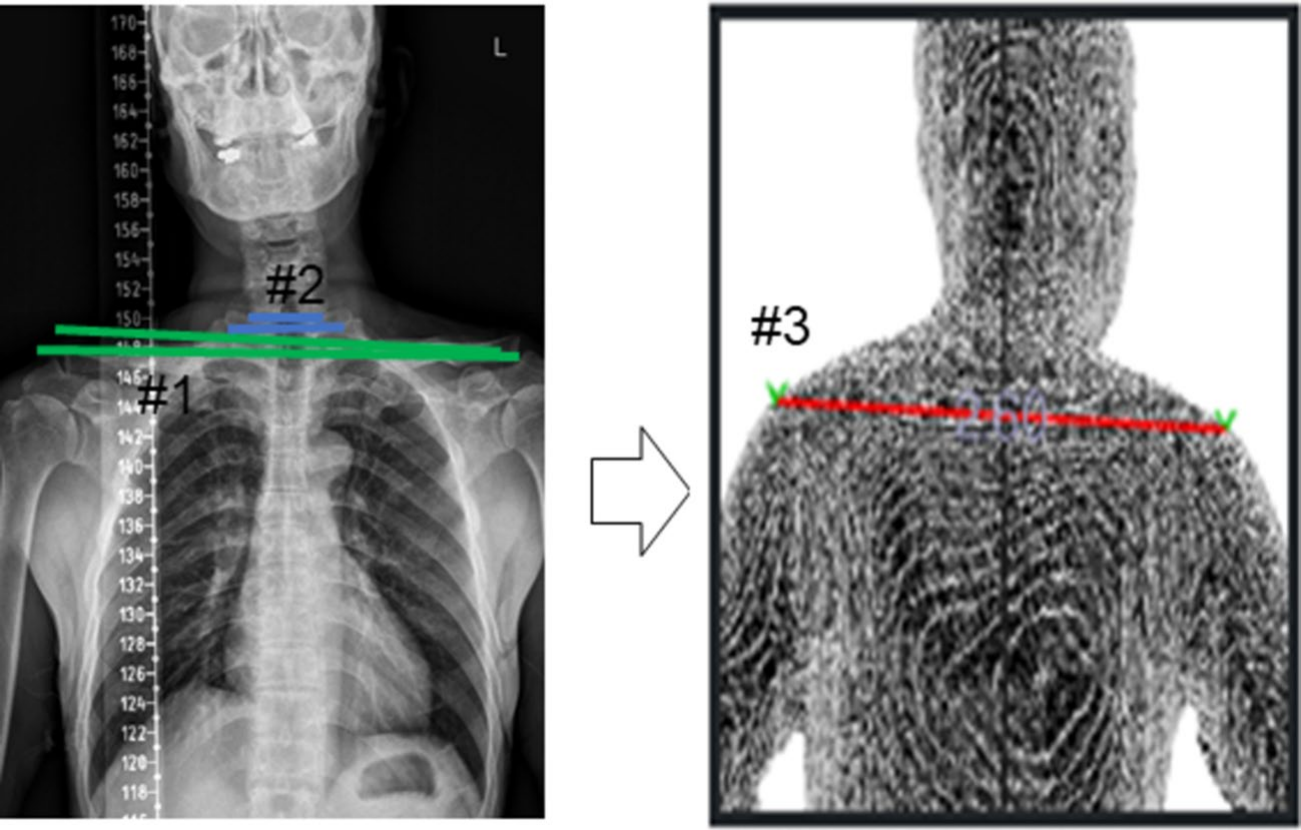
#1. Clavicle angle / #2. T1 coronal tilt on x-ray / #3. Shoulder gradient on the 3D scanner

The shoulder gradient in the coronal plane was correlated with the clavicle angle on the X-ray, and the C.C. and *p*-value were 0.373 and < 0.001. The correlation between the shoulder gradient and T1 coronal tilt exhibited lesser C.C. and higher *p*-value, which were 0.245 and 0.016, respectively.

### Correlation coefficient for the neck flexion, hip flexion, and knee flexion on 3D scanner

At first, we assumed that the aABC, aBCD, and aCDE would reflect neck flexion, hip flexion and knee flexion respectively (Fig. 1a). However, the aABC did not show any correlation with cervical sagittal parameters. The aBCD did not show a statistically significant correlation with the lumbopelvic parameters, including sacral slope or pelvic tilt. On the other hand, aBCD was found to be associated with SVA, and the C.C. and *p*-value were −0.260 and 0.010. Unfortunately, aCDE couldn’t be compared with similar knee flexion parameters of x-ray because whole spine x-ray images don’t contain the knees. However, aCDE was found to be associated with SVA, as reflected by a C.C. of 0.296 and a *p*-value of 0.003 and with lumbar lordosis, as reflected by a C.C. of −0.305 and a *p*-value of 0.002.

All the correlation coefficients and *p*-values from the result 3.1 ~ 3.5 are listed in Table 3.

**Table 3:** Correlation coefficient between scanner and x-ray parameters

Sagittal scanner parameter	X-ray parameter	
*Cervical parameter*	*C2-7 SVA*	*T1 slope*
aAB_sag	0.496(*p* < 0.001)	0.281(0.005)
dAB_hor	0.478(< 0.001)	0.276(0.006)
*Global parameter(thoraco-lumbar)*	*SVA*	
aBC_sag	0.222(0.029)	
dBC_hor	0.223(0.028)	
*Global parameter(cervico-thoraco-lumbar)*	*ODHA*	
aAC_sag	0.336(< 0.001)	
dAC_hor	0.335(< 0.001)	
*Neck flexion parameter*	*C2-7 SVA*	*SVA*
aABC	N.C	N.C
*Hip flexion parameter*	*Pelvic parameter* *(PI, PT, SS)*	*SVA*
aBCD	N.C	−0.260(0.010)
*Knee flexion parameter*	*SVA*	*Lumbar lordosis*
aCDE	0.296(0.003)	−0.305(0.002)
*Coronal scanner parameter*	*Clavicle angle*	*T1 coronal tilt*
Shoulder gradient (Lt. +)	0.373(< 0.001)	0.245(0.015)

*aAB_sag* Angle of AB with respect to plumb line, kyphosis + , *dAB_hor* Horizontal distance between A and B, kyphosis + ,* aABC* Angle of ABC, neck extension + , *aBCD* Angle of BCD, hip extension + , *aCDE* Angle of CDE, knee flexion +

### The effect of body composition data between x-ray and 3D scanner

About the cervical sagittal balance parameters, C2-7 SVA and dAB_hor show some correlation. (C.C. 0.478, *p*-value < 0.001). We applied a partial correlation coefficient (P.C.C.) adjusted the effect of body composition factors including BMI, PBF, SMI and all together. The P.C.C. adjusted the BMI score was 0.475, with a *p*-value of < 0.001. The P.C.C. adjusted the PBF score was 0.390, with a *p*-value of < 0.001. The P.C.C. adjusted the SMI score was 0.354, with a *p*-value of < 0.001. The P.C.C. adjusted the BMI, PBF, and SMI togethers were 0.312, with a *p*-value of < 0.003, which shows less relationship than the previous one.

About the thoracolumbar sagittal balance parameters, SVA and dBC_hor show some correlation. (C.C. 0.223, *p*-value 0.028). Meanwhile, the data adjusted the BMI score were 0.226 and 0.028. The data adjusted the PBF score were 0.234 and 0.023, and the data adjusted the SMI score were 0.220 and 0.034. The data adjusted the BMI, PBF, and SMI scores were 0.272 and 0.009, which shows not that different from the previous one.

About the global sagittal balance parameters, ODHA and aAC_sag show some correlation. (C.C. 0.336, *p*-value < 0.001). The data adjusted the BMI score were 0.339 and < 0.001. The data adjusted the PBF score were 0.330 and 0.001. The data adjusted the SMI score were 0.301 and 0.003. The data, adjusted the BMI, PBF, and SMI scores, were 0.342 and < 0.001, which shows not that different from the previous one.

About the coronal balance parameters, Clavicle angle and shoulder gradient show some correlation. (C.C. 0.373, *p*-value < 0.001). The data adjusted the BMI score were 0.362 and < 0.001. The data adjusted the PBF score were 0.379 and < 0.001. The data adjusted the SMI score were 0.376 and < 0.001. The data, adjusted the BMI, PBF, and SMI scores, were 0.366 and < 0.001, which shows not that different from the previous one.

In short, the body composition factors didn’t affect the correlation between the two parameters well (Table 4).

**Table 4:** Partial correlation coefficients adjusted the body composition factors

	C.C.(*p*-value)	Adjusted BMI	Adjusted PBF	Adjusted SMI	Adjusted BMI, PBF, and SMI
C2-7 SVA & dAB_hor	0.478(< 0.001)	0.475(< 0.001)	0.390(< 0.001)	0.354(< 0.001)	0.312(0.003)
SVA & dBC_hor	0.223(0.028)	0.226(0.028)	0.234(0.023)	0.220(0.034)	0.272(0.009)
ODHA & aAC_sag	0.336(< 0.001)	0.339(< 0.001)	0.330(0.001)	0.301(0.003)	0.342(< 0.001)
Clavicle angle & shoulder gradient	0.373(< 0.001)	0.362(< 0.001)	0.379(< 0.001)	0.376(< 0.001)	0.366(< 0.001)

*C2-7 SVA *C2-7 Sagittal vertical axis, dAB_hor: Horizontal distance between B and C kyphosis + , *ODHA* Odontoid hip axis angle, aAC_sag: Angle of AC with respect to plumb line kyphosis + , *C.C.* Correlation coefficient, *BMI* Body mass index, *PBF *Percentage body fat, *SMI* Skeletal muscle index

To apply multiple linear regression analyses to 3D scanner data, the aAC_sag and dBC_hor were selected because they represent global sagittal balance and thoracolumbar sagittal balance respectively and they have similar X-ray parameters respectively (ODHA and SVA). The variables selected were gender, age, height, weight, BMI score, PBF score, SMI score, waist-hip ratio, and the fixed variable was ODHA for aAC_sag and SVA for dBC_hor, respectively. Before analysis, to avoid multicollinearity problems, the C.C. between variables was checked (Table 5).

**Table 5:** Correlation coefficients between demographic factors, body composition, and x-ray parameters

	BMI	PBF	SMI	Waist/hip ratio	Weight	Height	Age
ODHA	0.242 (0.018)	N.S	0.284 (0.005)	N.S	0.239 (0.020)	N.S	N.S
SVA	0.309 (0.002)	0.310 (0.002)	N.S	N.S	N.S	N.S	0.204(0.047)

*ODHA* Odontoid hip axis angle; *SVA* Sagittal vertical axis; *BMI* Body mass index; *PBF* Percentage body fat; *SMI* Skeletal muscle index

In terms of aAC_sag, ODHA served as the main explanatory variable. BMI, PBF, and SMI scores could affect aAC_sag independently. BMI score had a positive effect on positive global sagittal balance, but both SMI and PBF have a negative effect. According to the analysis of dBC_hor, SVA served as the main explanatory variable and the results were similar. In a simple regression analysis, the height was related to dBC_hor because dBC_hor is a factor related to the body length. BMI score had a positive effect on anterior thoracolumbar tilting. The PBF score and SMI score had a negative effect (Table 6).

**Table 6:** Multiple regression analysis (aAC_sag and dBC_hor for dependent variable and ODHA and SVA for fixed variable)

	aAC_sag&ODHA				dBC_hor&SVA			
	Simple linear regression		Multiple linear regression (fixed variable: ODHA)		Simple linear regression		Multiple linear regression (fixed variable: SVA)	
	Estimate (SE)	*P* value		Pr( >|t|)	Estimate (SE)	*P* value		Pr( >|t|)
x-ray data ODHA/SVA	0.49 (0.15)	0.001	0.46 (0.14)	0.002	0.30 (0.13)	0.03	0.36 (0.13)	0.008
Sex	1.56 (0.90)	0.09			−11.83 (7.58)	0.12	−21.31 (10.77)	0.051
Age	0.03 (0.05)	0.53			0.29 (0.39)	0.46		
Height(m)	0.01 (0.06)	0.86			−1.24 (0.52)	0.02		
Weight(Kg)	−0.02 (	0.67			−0.51 (0.38)	0.18		
BMI(Kg/m^2^)	−0.07 (0.14)	0.62	2.10 (0.45)	1.004 × 10–^05^	0.0076 (1.15)	0.99	18.27 (3.89)	9.665 × 10^–06^
Waist/hip ratio	−3.50 (2.91)	0.23			−34.30 (24.24)	0.16		
PBF	−0.14 (0.05)	0.01	−0.95 (0.18)	6.025 × 10–^07^	−0.12 (0.47)	0.81	−8.24 (1.58)	1.121 × 10^–06^
SMI	0.50 (0.51)	0.33	−6.09 (1.31)	1.199 × 10–^05^	−4.71 (4.26)	0.27	−51.30 (11.79)	3.607 × 10^–05^

*aAC_sag* Angle of AC with respect to plumb line kyphosis + ; *ODHA* Odontoid hip axis angle; *dBC_hor *Horizontal distance between B and C kyphosis + ; *SVA* Sagittal vertical axis; *BMI* Body mass index; *PBF *Percentage body fat; *SMI *Skeletal muscle index

### Clinical symptoms related to X-ray and 3D full body scanner parameters

Clinical symptoms are related to x-ray parameters in our study too. About the correlation between VAS and lumbar lordosis of x-ray, the C.C. and *p*-value were −0.220 and 0.031. About VAS score and sacral slope, the C.C. and *p*-value were 0.248 and 0.014. About mODI and lumbar lordosis, the C.C. and *p*-value were −0.283 and 0.005. About mODI and PI-LL mismatch, the C.C. and *p*-value were 0.270 and 0.007. And about mODI and the SVA, the C.C. and *p*-value were 0.215 and 0.034.

However, no 3D scanner sagittal parameter was related to clinical symptoms except aBCD and aCDE. The C.C. and *p*-value between aBCD and mODI score were −0.205 and 0.044 and C.C. and *p*-value between aCDE and mODI score were 0.245 and 0.015. Because aBCD was related with SVA and aCDE was related with SVA and loss of lumbar lordosis too. The partial correlation coefficient adjusted the SVA between aBCD and mODI score showed no statistical significance. The partial correlation coefficient adjusted the SVA and lumbar lordosis between aCDE and mODI score showed no statistical significance too. As a result, the mODI score was related to the loss of lumbar lordosis and positive sagittal balance (SVA), and aBCD and aCDE of the 3D full body scanner were related to the mODI score because of their correlations with positive sagittal balance and loss lumbar lordosis (Table 7).

**Table 7:** Correlation coefficient between parameters and clinical data

	C.C(*p*-value)	
x-ray parameter	VAS score	mODI score
Lumbar lordosis	−0.220(0.031)	−0.283(0.005)
Sacral slope	−0.248(0.014)	−0.253(0.012)
PI-LL mismatch	N.S	0.270(0.008)
SVA	N.S	0.215(0.034)
3D scanner parameter		
aBCD	N.S	−0.205(0.044)
aCDE	N.S	0.245(0.015)
3D scanner adjusted SVA effect	Partial C.C.(*p*-value)	
aBCD adjusted SVA	N.S	N.S
aCDE adjusted SVA and lumbar lordosis	N.S	N.S

*C.C *Correlation coefficient; *VAS* Visual analog scale; *mODI *modified Oswestry disability index; *PI-LL* mismatch Pelvic incidence – Lumbar lordosis mismatch; *aBCD* Angle of BCD, hip extension + ; *aCDE* Angle of CDE, knee flexion + ; *SVA* Sagittal vertical axis

## Discussion

This was a preliminary study to measure the sagittal and coronal balance of the adult spine using a new 3D full-body scanner. As a preliminary study, this study has many things that need to be improved, but this study showed a new possibility by having distinct points from previous 3D scanner studies of the spine balance. There are three different points with previous 3D scanner studies for spine evaluation.

First one is, as concerned, the evaluation of sagittal and coronal balance of adult spines, especially positive sagittal balance. Spine surgeons have been interested in the evaluation of positive sagittal balance since 2000 [12, 35]. But previous 3D scanner studies focused on the scoliosis evaluation using torso contour derived from a transverse plane. Because they checked spinous processes and to figure out the rotation of the vertebral body, which is an important phenomenon of scoliosis [13]. Of course, they could evaluate thoracolumbar kyphosis using the position of spinous processes in the sagittal and coronal planes. But by doing it, it has limitations of evaluating the upper cervical area or around hip joints, which are very important to cervical sagittal balance and global sagittal balance. So, we tried to create a new way to analyze the 3D full body scanner data.

Second one is that we selected a new AI algorithm to check inflection points of the sagittal plane and shoulder gradient of the coronal plane. The reason why we chose the points and shoulder gradient is that they are less affected by the soft tissues than the previous studies. The ears, lateral parts of shoulder, greater trochanters of femur, lateral parts of knee, lateral malleolus of ankle and acromio-clavicular joints are near the bony structures and less covered by the soft tissue. The patients of degenerative spondylosis have various BMI and different soft tissue status and it is difficult to analyze their spinal balance by using body contours only and we chose to select those points.

Last one is that it doesn’t use any adhesive markers [5]. Adhesive markers could result in some unwanted contacts, which results in discomfort to patients [9].

Although the correlations between 3D full body scanner data and x-ray data were statistically significant, the correlation coefficient levels were weak and moderate. At first, we thought that the discrepancy could be due to the interferences of soft tissue like fat tissue. But in our data, according to the partial correlation coefficient, it wasn’t due to soft tissue problem. And, interestingly, the C.C. of thoracolumbar sagittal balance was smaller than that of cervical sagittal balance. There are some reasons about the weak to moderate correlations.

The first one is the positioning difference. According to our scanner design, the participants should stand with both hands on the handle even though they didn’t lean their upper body. But during x-ray scan, the hands were placed on the shoulders to prevent both arms blocking their vertebrae. According to previous study, the discrepancy of positioning could lessen the C.C especially below the shoulder level [30].

**Fig. 6:**
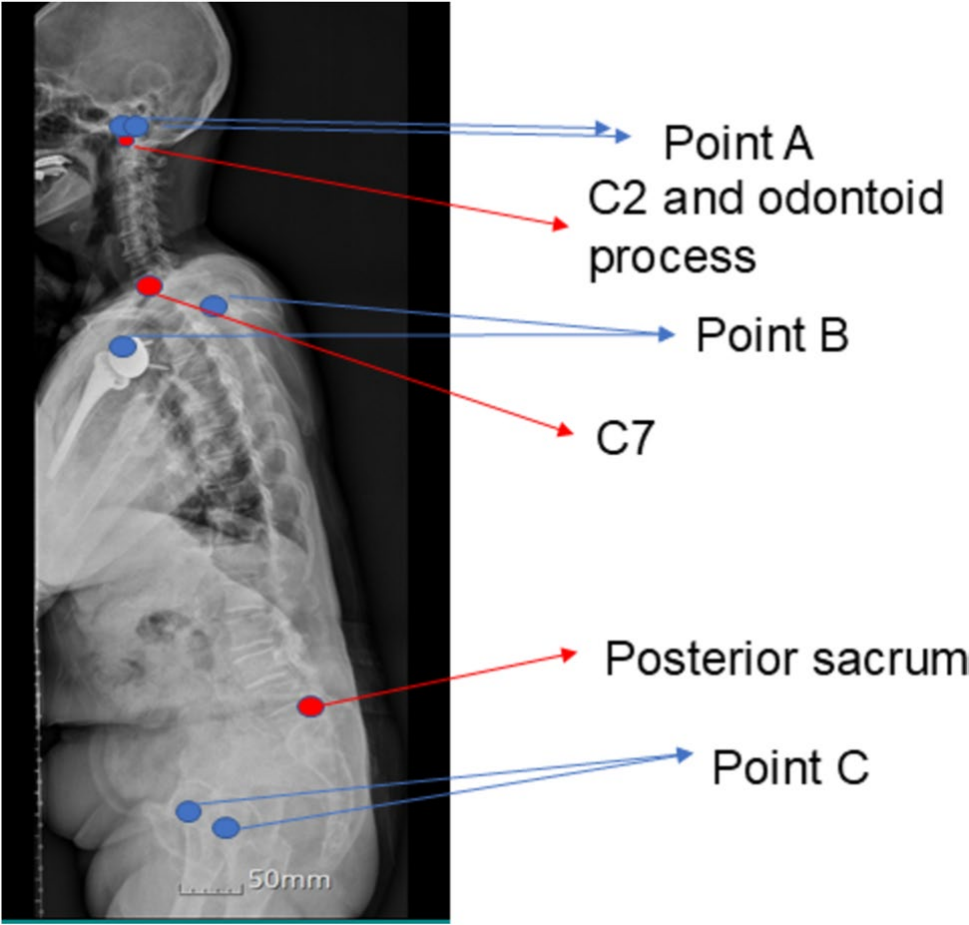
Point A,B,C – 3D scanning point / C2 and odontoid process, C7, posterior sacrum – x-ray point

Second, there are inevitable differences between the bony structures and the 3D scanner inflection points. Point A is near the C2 vertebra in x-ray and point B is less but relatively near the C7 vertebral body too. However, point C is far from the posterior sacrum (Fig. 6). There was an inevitable discrepancy around the hip joint. Consequently, there were weak correlation level in the data of point C, like thoracolumbar sagittal parameters. Our next study will focus on how to minimize the point difference between the 3D scanner and X-ray.

Third one is the multiple averaging procedures during every step. We did average procedures during x-ray evaluation, multiple 3D scanning data and even integration of left and right data. The repeated average procedures might lessen the correlation coefficients.

**Fig. 7:**
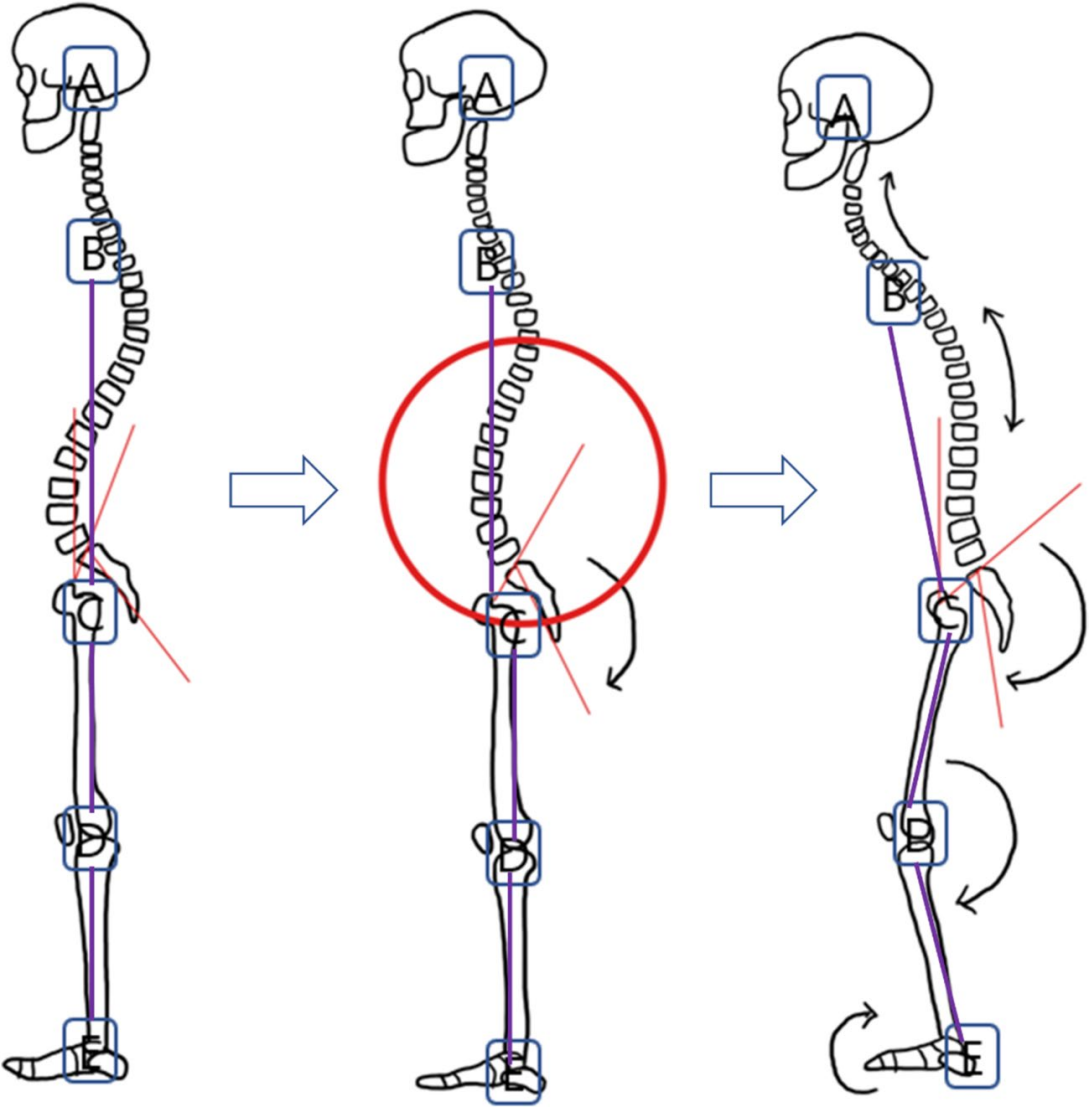
The compensatory pelvic retroversion and knee flexion mechanism. Loss of lumbar lordosis can be compensated by the pelvic retroversion. (Red circle) But aBCD and aCDE are not changed. (middle one). More loss of lumbar lordosis cannot be compensated by the pelvic retroversion and additional compensatory knee flexion tried to reduce sagittal vertical axis. aBCD and aCDE are changed (Right one)

In our data, aBCD showed a correlation with thoracolumbar kyphosis and aCDE showed a correlation with thoracolumbar kyphosis and lumbar lordosis. We thought this is related to the compensatory mechanism of hip and knee flexion to positive sagittal balance. Many studies said that the mild decrease of lumbar lordosis due to spondylosis can be compensated by the pelvic retroversion, which leads to normal SVA. But much more decrease of lumbar lordosis can’t be compensated by the pelvic retroversion because of its limitation and additional compensation can be achieved by the compensatory knee flexion [2, 26] (Fig. 7).

The aBCD is different with hip flexion because the point B is around the shoulder, so aBCD didn’t show correlation with spinopelvic parameters like pelvic tilt or sacral slope and showed correlation with thoracolumbar kyphosis. And aCDE, similar with knee flexion, showed the correlation with positive sagittal balance and lumbar lordosis. Compensatory mechanism of knee flexion should be considered in ASD analysis but the whole spine x-rays don’t contain knee parts. Meanwhile, our 3D full body scanner caught the mechanism. And also, this phenomenon resulted in the correlations between the 3D scanner data “aBCD, aCDE” and mODIs reflecting clinical symptoms too.

As concerned, according to partial correlation coefficients, the obesity and body composition itself didn’t affect the correlation between scanner and x-ray parameters. This might be related to some reasons. First, All the participants were East Asians and there are no severe obese patients more than 35 BMI scores. And second, the obesity pattern of them tends to be abdominal obesity [27]. But in our 3D scanner, the inflection points are shoulder and hip area, which are not affected by abdominal obesity. And of course, obesity itself affects both 3D scanners and x-ray parameters simultaneously, which results in diminishing the effect of partial correlation coefficient. At last, as concerned, the inflection points and shoulder gradients were chosen to lessen the effects of obesity.

To avoid multicollinearity problems during multiple regression study, we calculated the correlation between body composition and x-ray parameters and the obesity was related to positive sagittal imbalance, which is similar with previous studies showing obese patients have a difficulty of compensatory pelvic retroversion to deal with sagittal malalignment [1, 3, 18]. In addition, the age was related with SVA but not with ODHA, which is like the previous studies [8]. SMI score was related to ODHA, which is hard to explain, we concluded that it is due to confounding between BMI, PBF and SMI.

And according to multiple regression studies, BMI, PBF, and SMI scores affected 3D scanner parameters independently. Especially, BMI score had a positive effect on positive global sagittal balance independently with bony structures, which means heavyweight contrast to their own height could affect the anterior tilting during the 3D scanning. We thought our 3D scanner caught the gradual anterior tilting during the scanning due to long scan time. Previous studies showed that x-ray parameters of sagittal imbalance could be different after the patient stood a while or walked a while [20]. This is a concept of dynamic spine balance. In our 3D scanner, the scanning took 2 min and rotated the participant, which caused some fatigue of standing and balancing and resulted in the change of spinal balance. About dynamic spine balance, the obesity was an important factor for anterior tilting and high BMI was a risk factor for progressive worsening of dynamic sagittal imbalance [18, 20, 31]. And our obese participants tended to feel fatigue during scanning and stand anterior tilting, leading to the result of multiple regression analyses.

This device contains only 3 camera modules and the rotating bases system helps this scanner doesn’t take up a lot of space too. Also, the interpretation system of inflection points is based on AI algorithm. Those characters made this device’s costs only 25,000 U.S. dollars. Even though, this device and analyzing system couldn’t replace the need of x-ray or EOS system, considering the price of EOS system is 950,000 U.S. dollars [19], this device and analyzing system could be a good option with low costs. But as a preliminary study, it is too early to evaluate its cost-effectiveness.

This study, as a preliminary study, has several limitations. First, as mentioned, the number of participants was small as a preliminary study. The next study will include more participants. And second, there are some adjustable things like position difference between x-ray and 3D scanner. We will change the position change of 3D scanning. A hand grip should be removed and we prepare the next study of not rotating base of scanner.

Most importantly, there were several problems related to AI induced inflection points. At first, we only focused some inflections points less affected by the soft tissue and less deviation. This led to the inflection points at the hip and shoulder, but no points of detailed thoracolumbar spines. Limited inflection points at the hip and shoulder caused the limited interpretation of lumbar lordosis and detailed parameters of thoracolumbar spines. Due to it, there were less correlations between lumbar lordosis and 3D scanner data. This led the difficulty of reflecting the Roussouly classification which is very important to understand the adult spine deformity [32]. And also due to the same reason, our 3D scanner doesn’t accurately reflect minor deformations that compensate for sagittal vertical axis (SVA) changes. However, in adult spinal deformity cases involving pathological changes such as vertebral fractures, the quality of soft tissues, including paraspinal muscles, plays a critical role in the vertebral fracture cascade, ultimately contributing to the progression of deformities [22]. Considering these, some strategies are needed like creating new proper inflection points inside the thoracolumbar spines but less covered by soft tissues. And also, we will correct the AI human algorithm to catch the detailed data around thoracolumbar spines and add new inflection points.

Moreover, due to the weak to moderate correlation, the R^2^ of multiple regression analysis was negligible. So, the interpretation of causality had its own limitations. The next study will try to increase the correlations and it can help the interpretation of causality too. Finally, there needed to be more analyses on coronal balance. The subsequent study will cover a more detailed analysis of it.

## Conclusion

In our study, a new 3D full body scanner showed some correlations with x-ray parameters about the sagittal and coronal spine balance for adult spine. It has some different points with other previous 3D scanner studies analyzing the spine balance. Even though the correlation coefficient levels were weak and moderate, it has some strengths like radiation-free method, correlation with clinical symptoms, independence to soft tissue coverage, possibility of analyzing dynamic spine balance and low cost. As a preliminary study, it needs more corrections but has a possibility as new alternative to evaluate the adult spine balance.

## Data Availability

No datasets were generated or analysed during the current study.

## Abbreviations

ASD: Adult spine deformity
SVA: Sagittal vertical axis
AIS: Adolescent idiopathic scoliosis
AP: Anteroposterior
ODHA: Odontoid hip axis angle
PI: Pelvic incidence
PT: Pelvic tilt
SS: Sacral slope
LL: Lumbar lordosis
PI-LL mismatch: Pelvic incidence-Lumbar lordosis mismatch
VAS: Visual analog scale
mODI: Modified Oswestry Disability Index
BMI: Body mass index
PBF: Percentage body fat
SMI: Skeletal muscle index
aAB_sag: The angle of two points(A,B) with respect to the plumb line in the sagittal plane
dAB_hor: The horizontal distance between two points(A,B) in the sagittal plane
aABC: The angle of three points(A,B and C) in the sagittal plane
ICC: Intraclass correlation coefficient
CC: Correlation coefficient
